# Region-specific deficits in dopamine, but not norepinephrine, signaling in a novel A30P α-synuclein BAC transgenic mouse^☆^

**DOI:** 10.1016/j.nbd.2013.10.005

**Published:** 2014-02

**Authors:** Tonya N. Taylor, Dawid Potgieter, Sabina Anwar, Steven L. Senior, Stephanie Janezic, Sarah Threlfell, Brent Ryan, Laura Parkkinen, Thierry Deltheil, Milena Cioroch, Achilleas Livieratos, Peter L. Oliver, Katie A. Jennings, Kay E. Davies, Olaf Ansorge, David M. Bannerman, Stephanie J. Cragg, Richard Wade-Martins

**Affiliations:** a Oxford Parkinson's Disease Centre, University of Oxford, Oxford, UK; b Department of Physiology, Anatomy, and Genetics, University of Oxford, Oxford, UK; c Nuffield Department of Clinical Neuroscience, John Radcliffe Hospital, University of Oxford, Oxford, UK; d MRC Functional Genomics Unit, University of Oxford, Oxford, UK; e Department of Experimental Psychology, University of Oxford, Oxford, UK

**Keywords:** Parkinson's disease, α-Synuclein, Dopamine, Norepinephrine, Voltammetry, Behavior

## Abstract

Parkinson's disease (PD) is a neurodegenerative disorder classically characterized by the death of dopamine (DA) neurons in the substantia nigra pars compacta and by intracellular Lewy bodies composed largely of α-synuclein. Approximately 5–10% of PD patients have a familial form of Parkinsonism, including mutations in α-synuclein. To better understand the cell-type specific role of α-synuclein on DA neurotransmission, and the effects of the disease-associated A30P mutation, we generated and studied a novel transgenic model of PD. We expressed the A30P mutant form of human α-synuclein in a spatially-relevant manner from the 111 kb *SNCA* genomic DNA locus on a bacterial artificial chromosome (BAC) insert on a mouse null (*Snca −/−*) background. The BAC transgenic mice expressed α-synuclein in tyrosine hydroxylase-positive neurons and expression of either A30P α-synuclein or wildtype α-synuclein restored the sensitivity of DA neurons to MPTP in resistant *Snca −/−* animals. A30P α-synuclein mice showed no Lewy body-like aggregation, and did not lose catecholamine neurons in substantia nigra or locus coeruleus. However, using cyclic voltammetry at carbon-fiber microelectrodes we identified a deficit in evoked DA release in the caudate putamen, but not in the nucleus accumbens, of *SNCA*-A30P *Snca −/−* mice but no changes to release of another catecholamine, norepinephrine (NE), in the NE-rich ventral bed nucleus of stria terminalis. *SNCA*-A30P *Snca −/−* mice had no overt behavioral impairments but exhibited a mild increase in wheel-running. In summary, this refined PD mouse model shows that A30P α-synuclein preferentially perturbs the dopaminergic system in the dorsal striatum, reflecting the region-specific change seen in PD.

## Highlights

• *SNCA* A30P BAC transgenic mice recapitulate endogenous α-synuclein expression pattern
• *SNCA* A30P BAC transgenic mice demonstrate a region specific deficit in evoked DA, but not NE, release.
• Expression of A30P or WT α-syn restored the sensitivity of DA neurons to MPTP.
• A30P BAC mice had no Lewy body-like aggregation or neuronal loss in SNpc or LC.
• Early changes in DA neurotransmission in the absence of aggregation

## Introduction

Parkinson's disease (PD) is a debilitating neurodegenerative disease, second in prevalence to Alzheimer's disease. PD is primarily considered a dopaminergic disorder, characterized by motor phenotypes, including resting tremor, bradykinesia, rigidity and postural instability (Dauer and Przedborski, 2003, Olanow and Tatton, 1999). *Post-mortem* PD brain tissue shows loss of midbrain dopamine (DA) neurons and accumulation of Lewy bodies, protein aggregates largely composed of α-synuclein. The onset of classical parkinsonism is frequently preceded by a prodromal phase, with symptoms ranging from sleep disturbances to gastrointestinal dysfunction (Braak et al., 2003, Langston, 2006). These non-motor changes likely arise from changes in neurotransmission in non-dopaminergic systems.

Approximately 5–10% of PD cases are familial and rare dominant familial PD has been linked to the point mutations A53T, E46K, and A30P in α-synuclein (*SNCA*) on chromosome 4 (Kruger et al., 2001, Polymeropoulos et al., 1997, Zarranz et al., 2004). Recent *post-mortem* neuropathological analysis of a PD patient with an A30P α-synuclein mutation showed strong neuropathological similarities between A30P and idiopathic PD patients (Seidel et al., 2010), confirming the relevance of the mutation to PD. The function(s) of α-synuclein, a 140 amino acid protein highly abundant in presynaptic terminals, are incompletely resolved. In PD, α-synuclein accumulation and Lewy body formation occur early in structures highly prone to neurodegeneration (Braak et al., 2002, Goedert, 2001, Spillantini et al., 1997). However, α-synuclein aggregation is not required for neuronal loss (Fernagut and Chesselet, 2004).

Despite relatively few familial PD patients, genetic-based animal models have been integral to furthering knowledge about PD. Historically, genetic models of α-synuclein dysfunction have yielded a fatal motor syndrome, often in the absence of overt dopaminergic alterations. These models have had significant limitations due to the use of promoters that do not reproduce the endogenous pattern of neuronal gene expression (Chesselet, 2008). Furthermore, α-synuclein, either mutated and/or overexpressed, can damage neurons; however, the resulting degeneration may have limited relevance to PD (Sulzer, 2010).

To better understand the cell-type specific role of α-synuclein on DA neurotransmission, and the non-toxic physiological effects of the disease-associated A30P mutation, we have generated and studied a novel transgenic model of PD. We used a bacterial artificial chromosome (BAC), containing the complete 111 kb *SNCA* genomic DNA locus to generate transgenic mice which express either human α-synuclein carrying the PD-associated A30P mutation or human wild-type α-synuclein as a control, on an α-synuclein knockout (*Snca −/−*) background. Expression of either A30P α-synuclein or wildtype α-synuclein restored the sensitivity of dopaminergic neurons to MPTP in resistant *Snca −/−* animals. We demonstrate here that the *SNCA-A30P Snca −/−* mice exhibit a deficit in DA neurotransmission in the dorsal, but not ventral, striatum as shown by fast-scan cyclic voltammetry (FCV), suggesting a specific role for α-synuclein in the nigrostriatal system preferentially vulnerable in PD. To investigate the role of α-synuclein and the effect of the A30P mutation on neurotransmission of another catecholamine, norepinephrine, we developed an FCV assay to explore norepinephrine neurotransmission in the ventral bed nucleus stria terminalis (vBNST), but we find no significant changes in the *SNCA-A30P Snca −/−* line. These mice provide a novel model to study differential region-specific effects of α-synuclein on dopamine neurotransmission.

## Materials and methods

### Breeding

Male and female mice lacking α-synuclein (*Snca*−/−) were maintained as a pure-bred C57BL6 background (Abeliovich et al., 2000). Bacterial artificial chromosomal (BAC) DNA containing *SNCA* transgenes encoding either the wild-type *SNCA* locus (Peruzzi et al., 2009) or the A30P mutation engineered using standard BAC recombineering methods were prepared by CsCl double banding for microinjection into pure C57/Bl6 (Charles River, Margate, UK) mouse pronuclei. Founder pups were screened for the presence of the intact transgene by PCR and breeding lines were established. The resulting lines were backcrossed for a minimum of six generations to C57/Bl6 *Snca −/−* mice to obtain > 99% pure C57/Bl6 background, resulting in *SNCA-A30P + Snca −/−* (referred to as A30P) and *SNCA-WT + Snca −/−* (referred to as hα-syn) mice. All transgenic lines were maintained as hemizygotes. All animal work was performed in accordance with the United Kingdom Animals (Scientific Procedures) Act (1986).

### Polymerase chain reaction (PCR)

Genotyping was carried out to identify the mice carrying the BAC-*SNCA* transgenes. PCR was used to amplify the wild-type or knock-out *Snca −/−* alleles, and to amplify the junctions between the BAC vector and the *SNCA* genomic DNA insert. Exon PCR was used to confirm all six exons from the BAC inserts were present. Separate master mixes were prepared for each primer pair. The primer sequences used were previously designed in our laboratory and are listed in Table 1. PCR amplification conditions were denaturing for 15 min at 95 °C, followed by 44 cycles of 30 s at 95 °C, 30 s at 60 °C, and 1 min at 72 °C (35 cycles for exon PCR).

**Table 1:** List of primer sequences used for PCR. All PCR primer sequences are given 5′– 3′.

Name	Forward primer	Reverse primer	Product size
Mouse *Snca* WT	CAGCTCAAGTTCAGCCACGA	AAGGAAAGCCGAGTGATGTAC	485 bp
Mouse *Snca* KO	CAGCTCAAGTTCAGCCACGA	ATGGAAGGATTGGAGCTACG	550 bp
BAC-*SNCA*	CACTGAGTATTGTTCTGGTAAC	TCAAACATGAGAATTGGTCG	450 bp
5 junction			
BAC-*SNCA*	GGCCTCTGTCGTTTCCTTTCTCTG	ACGTGCATAGGCAGAGCTAAA	450 bp
3′ junction		ACCT	
*SNCA* Exon 1	ATCCGAGATAGGGACGAGGAG	AATAGGAAAGAAGAAGGAAAA	465 bp
		GGAG	
*SNCA* Exon 2	CCGAAAGTTCTCATTCAAAGTG	TGACATTTGGGGTTTACCTACC	206 bp
*SNCA* Exon 3	GAAAACTAGCTAATCAGCAATTTAA	AATCTTGAATACTGGGCCACAC	182 bp
	GG		
*SNCA* Exon 4	CCACCCTTTAATCTGTTGTTGC	CACAAAACGTACACAGCCATA C	203 bp
*SNCA* Exon 5	TCATCATGTTCTTTTTGTGCTTC	TGTGACAATGACAGGTTTTTGG	182 bp
*SNCA* Exon 6	AACAGTGTGTGCTGTCTTTTTG	AGATTGAAGCCACAAAATCCA	317 bp
		C	

### Fluorescence in situ hybridization (FISH)

The protocol used for extraction of primary fibroblasts was modified from Kulnane and colleagues (Kulnane et al., 2002). FISH was performed using the *SNCA* BAC as a probe on primary fibroblasts as previously described (Jefferson and Volpi, 2010).

### MPTP treatment in mice

Animals (male and female) were injected intraperitoneally three times, spaced two hours apart, with either saline or 15 mg/kg of the dopaminergic neurotoxin MPTP (free base). Animals were sacrificed 7 days after MPTP administration for analysis of dopaminergic markers.

### Western immunoblotting analysis

Western blots were used to quantify the amount of tyrosine hydroxylase (TH), dopamine transporter (DAT), human α-synuclein, and actin present in samples of striatal tissue and were performed as described previously (Caudle et al., 2006). Membranes were incubated overnight in a TH monoclonal antibody (1:1000; Chemicon) and detected using a goat anti-rabbit horseradish peroxidase secondary antibody (1:5000) and visualized by enhanced chemiluminescence using an ECL kit (GE). Membranes were stripped for 15 min at room temperature with Restore Stripping Buffer (Thermo Fisher, Rockford, IL, USA) and sequentially re-probed with DAT (1:5000; Chemicon), and human α-synuclein (1:500; Abcam) antibody. Actin blots were used to ensure equal protein loading across samples.

For MPTP sample analysis, striata were mechanically homogenized in RIPA buffer and cytosolic fractions taken prepared for reducing, denaturing SDS-PAGE. Proteins were separated on 10.5–14.5 % gradient gels and transferred using a Trans-Blot Turbo transfer system (Bio-Rad, UK). Membranes were blocked and probed with polyclonal rabbit anti-tyrosine hydroxylase (Millipore, AB152) or polyclonal rabbit anti β-actin antibody (Abcam, AB8227) and visualized with HRP conjugated anti-rabbit IgG (Bio-Rad, UK). Densitometry analysis was performed using ImageJ software.

### Immunohistochemistry

Tissue staining was completed as previously described (Caudle et al., 2006, Miller et al., 1997, Miller et al., 1999). Briefly, A30P, hα-syn, and *Snca*−/− mice were perfused transcardially with phosphate buffered saline (pH = 7.4) and then with 4% paraformaldehyde, removed, placed in 4% paraformaldehyde for 24 h, and finally cryoprotected in 30% sucrose for 48 h. The brains were then cut to a thickness of 40 μm a freezing microtome (Leica Microsystems). Sections were incubated with a polyclonal anti-TH (1:2000; Chemicon), monoclonal anti-DAT (1:750; Chemicon), or monoclonal anti-α-synuclein (1:500; BD Biosciences) antibody overnight and then incubated in a biotinylated goat anti-rabbit, goat anti-rat, or goat ant-mouse secondary antibody for 1 h at room temperature. Visualization was performed using 3, 3´-diaminobenzidine (DAB) for 45 s at room temperature. After DAB, all sections were mounted on slides, dehydrated, and coverslipped using DPX Mountant (Fluka Biochemika) and sections were viewed using a light microscope (Leica).

To detect potential α-synuclein-related pathology, we used immunohistochemical staining by monoclonal antibodies (MAb) Syn-1 (1:1000, BD Transduction Labs) against aa 91–99, LB509 (1:1000, Invitrogen) against amino acids (aa) 115–122 of α-syn and pSyn#64 (1:5000, Wako) against aa124–134 (including phosphorylated Ser129) on paraffin sections. Other antibodies included MAb anti-TH (1:100, Millipore); anti-Iba-1 (1:2000, WAKO); polyclonal anti-p62 (1:5000, Enzo) and anti-GFAP (1:4000, Dako). The fixed brain and spinal cord were embedded in paraffin and cut into 6 μm-thick sections, deparaffinised and rehydrated. Peroxidase activity was eliminated with treatment with 3% H_2_O_2_ followed by pre-treatments with formic acid for 15 min (for Syn-1), formic acid for 5 min together with autoclaving (121 °C) for 10 min in citrate buffer (for LB509, Iba-1, p62 and TH), 20 μl/ml proteinase K (Roche, UK) treatment for 10 min (for pSyn#64), microwaving in citrate buffer for 3 × 5 min (for GFAP). The mouse-on-mouse kit (M.O.M., Vector Labs) was used to MAb to minimize the background and staining was visualized using DAB (Vector Labs).

### Fast scan cyclic voltammetry

#### Brain slice preparation

Adult *Snca*−/−; A30P and hα-syn mice (aged 3–4 months) were sacrificed by cervical dislocation and decapitated; their brains were removed over ice. Coronal brain slices (300 μm thick) containing the striatum or the ventral nucleus of the bed nucleus stria terminalis (vBNST) were prepared using a vibratome (Leica Microsystems) in ice-cold HEPES-based buffer saturated with 95% O_2_/5% CO_2_ as described previously (Cragg, 2003, Rice and Cragg, 2004). Slices were maintained at room temperature in HEPES-based buffer for ≥ 1 h before transfer to the recording chamber where slices were equilibrated for at least 30 min in the recording solution, a bicarbonate-buffered artificial CSF (containing 2.4 mm Ca^2 +^)(Cragg, 2003, Rice and Cragg, 2004).

#### Voltammetry

Extracellular catecholamines were monitored and quantified in the dorsal and ventral striatum at 32 °C using fast-scan cyclic voltammetry (FCV) as previously described (Rice and Cragg, 2004) with 10 μm-diameter carbon-fiber microelectrodes (exposed tip length, 50–100 μm, fabricated in-house) and a Millar voltammeter (Julian Millar, Barts and The London School of Medicine and Dentistry, London, UK). The applied voltage was a triangular waveform (− 0.7 to + 1.3 V range *vs.* Ag/AgCl) at a scan rate of 800 V/s and a frequency of 8 Hz, and was switched out of circuit between scans. Electrodes were positioned in striatal slices to a depth of 100 μm. Catecholamine release was evoked by a surface, concentric bipolar Pt/Ir electrode (25 μm diameter; FHC) as described previously (Rice and Cragg, 2004).

#### Striatal dopamine detection

Individual data points were collected through one of two experimental designs. In the first paradigm, evoked extracellular DA concentrations [DA]_o_ were assessed and compared across several sites per slice (6–8 sites per CPu, 2 per NAc) in both genotypes on the same experimental day. DA release data were collected from six different subdivisions, encompassing the dorsal and ventral CPu: Specifically, recordings were obtained from dorso-medial, dorso-central, dorso-lateral, central, ventro-medial and ventro-lateral regions. Stimuli in these experiments consisted of either a single pulse or burst pulses (4 pulses at 100 Hz). In the second stimulation paradigm, the frequency dependence of release was determined, through recordings taken repeatedly at given recording sites (2.5 min intervals ensured consistent release) and consisted of single pulses and trains of 5 pulses at a range of frequencies (1–100 Hz) applied in randomized order. These frequencies include the full range of dopaminergic neuron firing frequencies reported *in vivo*. Recordings in these experiments in CPu were in the dorsal half of the nucleus, and those in NAc were ventral to the anterior commissure and lateral to the lateral ventricle.

Stimulus pulses were generated out-of-phase with FCV scans and were applied at the lowest current that generated maximal dopamine release with a single stimulus pulse in wild-type animals (650 μA, 200 μs pulse duration). Evoked currents were attributable to DA by the presence of oxidation and reduction currents with peak potentials seen for DA in calibration media (+ 500–600 mV and − 200 mV *vs* Ag/AgCl, respectively).

DA uptake rates were compared between genotypes by comparing the decay phases of DA transient profiles evoked by a single pulse. DA uptake via DAT is the principal factor governing DA decay in these evoked transients (Giros et al., 1996). The rate of DA uptake by the DAT obeys Michaelis–Menton kinetics and is therefore proportional to *V_max_* and varies with [DA]_o_. Comparison of the decay phase of DA transients matched for similar peak [DA]_o_ eliminates differences in uptake rate due to [DA]_o_, therefore any prevailing differences in *V_max_* should be apparent (Cragg et al., 2000). DA transients were concentration-matched for a peak of 1.0 ± 0.01 μM from five animals for each genotype.

#### Norepinephrine detection in vBNST

Extracellular norepinephrine (NE) was monitored and quantified in the ventral bed nucleus of the stria terminalis (vBNST). Catecholamine detection using FCV has previously been described in vBNST (Miles et al., 2002) although the distinction between DA and NE had not been previously made. Evoked signals were readily attributable to catecholamines (NE/DA) by comparison of potentials for peak oxidation and reduction currents with those seen in calibration media post-experiment (1–2 μM) (approximately − 600 mV and − 200 mV *vs* Ag/AgCl, respectively). NE and DA have similar oxidation and reduction peak potentials, however, we attributed these signals to NE rather than DA, by identifying that these signals were regulated by α_2_ adrenergic receptors and by the NE transporter (NET) unlike DA signals detected in the adjacent striatum. Unless otherwise stated, individual data points were obtained from recordings taken repeatedly at a single recording site at 7 min intervals, to ensure consistent release (Miles et al., 2002) and consisted of trains of 30 pulses at a range of frequencies (10–100 Hz) in randomized order.

#### FCV analysis

Data were acquired and analyzed using Strathclyde Whole Cell Program (University of Strathclyde, Glasgow, UK) or Axoscope 10.2 (Molecular Devices). The number of animals in each data set is > 5. Comparisons for differences in means were assessed by one-way ANOVA and *post hoc* Bonferroni's multiple-comparison *t* test or unpaired *t* test using GraphPad Prism 4.0. For FCV data where absolute [DA]_o_ were compared across many striatal regions and slices between genotypes, all observations were treated as independent since data were obtained from different regions within a given striatum which show large variation due to striatal territory, a variation that is greater than that between slices.

#### HPLC

Tissue catecholamine contents were assessed from tissue punches by HPLC with electrochemical detection (ECD) as described previously (Al-Wandi et al., 2010, Senior et al., 2008) using a 4.6 × 150 mm Microsorb C18 reverse-phase column (Varian) and Decade II ECD with a Glassy carbon working electrode (Antec Leyden) set at + 0.7 V with respect to a Ag/AgCl reference electrode. For measuring dopamine and its metabolites, the mobile phase consisted of 12% methanol (v/v), 0.1 M monosodium phosphate, 2.4 mM 1-octane sulfonic acid (OSA), 0.68 mM EDTA, pH 3.1. For norepinephrine, a mobile phase of the same composition but containing 3.2 mM OSA was used.

#### Stereological analysis

Stereological sampling was performed using the Stereo Investigator software (MicroBrightField, Colchester, VT). Tissue staining was performed as described previously (McCormack et al., 2002, Reveron et al., 2002). Whole brains were removed and processed for frozen sections as described above, and serially sectioned at 50 μm for systematic analysis of randomly placed counting frames (size 50 × 50 μm) on a counting grid (size of 120 × 160 μm) and sampled using a 22 μm optical dissector with 2 μm upper and lower guard zones. The boundaries of substantia nigra pars compacta (SNpc) and locus coeruleus (LC) were outlined under magnification of the 10 × objective as per the atlas of Paxinos and Franklin (2001). Cells were counted with a 40 × objective (1.3 numerical aperture) using a Nikon Eclipse e800 microscope. Guard zones of 2 μm ensured the exclusion of lost profiles on the top and bottom of the section sampled. A dopaminergic or noradrenergic neuron was defined as an in-focus tyrosine hydroxylase immunoreactive (TH-IR) cell body with a TH-negative nucleus within the counting frame. For the SNpc, every other section was processed for TH-IR and counterstained with hematoxylin, resulting in 15 sections sampled per mouse. For the LC, every section was stained for TH-IR and counterstained with hematoxylin, resulting in 18–20 sections sampled per mouse. The number of neurons in the SNpc and LC was estimated using the optical fractionator method, which is unaffected by changes in the volume of reference of the structure sampled (West et al., 1991). Between 100 (LC) and 200 objects (SNpc) were counted to generate the stereological estimates. Gundersen (*m* = 1) coefficients of error were less than 0.1.

### Behavior

#### Locomotor activity

To measure novelty-induced locomotor activity, experiments were conducted in the light phase in an isolated behavior room between 800 and 1200 h. Ambulations were measured in transparent plexiglass cages placed into a rack with seven infrared photobeams (San Diego Instruments Inc., LaJolla, CA). Ambulations were recorded for 4 h (Schank et al., 2006). To measure circadian locomotor activity, mice were placed in the plexiglass cages and ambulations were recorded for two 24-h sessions of normal 12-h light/dark cycle (12:12 LD) and averaged.

#### Forepaw stride length

Prior to testing, animals were trained to walk across a clean sheet of paper into their home cage without stopping. Animals had their forepaws placed in black ink and the length of forepaw steps during normal walking (in a straight line only) was measured; animals were immediately placed back into their home cage upon completion of the task. Stride lengths were determined by measuring the distance between each step on the same side of the body, measuring from the middle toe of the first step to the heel of the second step and averaging the stride length per animal (Tillerson et al., 2002).

#### Rotarod

Animals were placed on an accelerating rotarod at the same time daily for 3 consecutive days, receiving 3 trials per day. The rotarod was accelerated from 4 to 40 revolutions per minute (RPMs) over a 5 min trial period. The latency to fall off the rotarod was recorded.

#### Circadian screen

Age-matched animals were individually housed in cages fitted with running wheels (Actimetrics) maintained at constant temperature and humidity, with *ad libitum* food and water. Cages were arranged in light-controlled chambers with externally controlled LED lighting, with light levels assessed at cage level using a lux meter. A behavioral screen was used to examine common circadian paradigms, consisting of: 1) 14 days entrainment under a 12-h 150 lux light/dark (12:12 LD) cycle (lights off at ZT12 where ZT0 = 0600), 2) 1 day negative masking using a 1 h light pulse at ZT14, 3) 14 further days of 12:12 LD, 4) 1 day 6-h phase advance, followed by 21 days of re-entrainment (12:12 LD, ZT0 = 0000), 5) 14 days under constant dark (DD) to measure free-running period, 6) 14 days under constant light (150 lux LL) and 7) Return to 12:12 LD ZT0 = 1200 for 14 days. Data analysis was carried out using 10 min bins of wheel running activity using Clocklab (Actimetrics) software.

#### Elevated plus maze

The elevated plus maze (EPM) paradigm was adapted from Schank and colleagues (Schank et al., 2008, Taylor et al., 2009). No drugs were administered prior to behavioral testing. Videotapes were later scored by an observer who was blind to genotype. The measure used for analysis is the percentage of time spent exploring the open arms, which was calculated by dividing the time spent in the open arms by the combined time spent in open and closed arms (Pellow et al., 1985). Data from male and female mice were combined, since there were no detectable sex differences.

#### Tail suspension test

These experiments were conducted using the methods of Cryan and colleagues (Cryan et al., 2004, Taylor et al., 2009). Mice were individually suspended by the tail to a horizontal ring stand bar (distance from the floor = 30 cm) using adhesive tape. A 5-min test session was videotaped and scored by a trained observer for escape-oriented behavior and bouts of immobility.

#### One hour stool collection

Each mouse was placed in a separate clean cage, without bedding, and observed throughout a 60 min collection period. Fecal pellets were collected immediately after expulsion and placed in sealed (to avoid evaporation) 1.5 ml tubes. Tubes were weighed to obtain the wet weight of the stool, this was then dried overnight at 60 °C and reweighed to obtain the dry weight and the stool water content (Li et al., 2006, Taylor et al., 2009).

#### Spontaneous alternation in a T-maze

Spontaneous alternation in an enclosed T-maze was performed as described previously (Deacon and Rawlins, 2006). The apparatus consisted of a black T-shaped wooden maze made of arms, measuring 30 cm in length, 10 cm in width, and 29 cm in height. The goal arm entrances were provided with sliding guillotine doors. A central partition wall, extending 7 cm into the start arm, divided the choice point into two goal arms to prevent the mouse from receiving any sensory input from the non-visited arm. A mouse was placed in the start arm of the T-maze and allowed to choose a goal arm. The mouse was then confined to the goal arm by sliding the guillotine door down. The arm entered (left or right) was recorded and the mouse was allowed to explore the goal arm for 30 s. The mouse was returned to the start arm, with the guillotine doors re-opened and the central partition removed, and allowed to explore again. The goal arm entered on the second run was recorded (left or right) and the mouse was then returned to its home cage. Each mouse was tested twice each day for 5 days (a total of 10 trials). The percentage of trials in which the mouse entered a different goal arm on the second run (*i.e.* alternated) was calculated. Data from male and female mice were combined, since there were no detectable sex differences.

#### Statistical analysis

Data from male and female mice were combined, since there were no detectable sex differences. All data were analyzed using one-way ANOVA (dot test, body weight, stool collection) completely randomized two-factor ANOVA followed by Bonferroni *post hoc* analysis (EPM, tail suspension test, T-maze, stride length, novelty-induced locomotor activity). Analyses were completed using Graph Pad Prism 4.0 for Windows.

## Results

### Generation of SNCA-WT Snca −/− and SNCA-A30P + Snca −/− mice

**Fig. 1:**
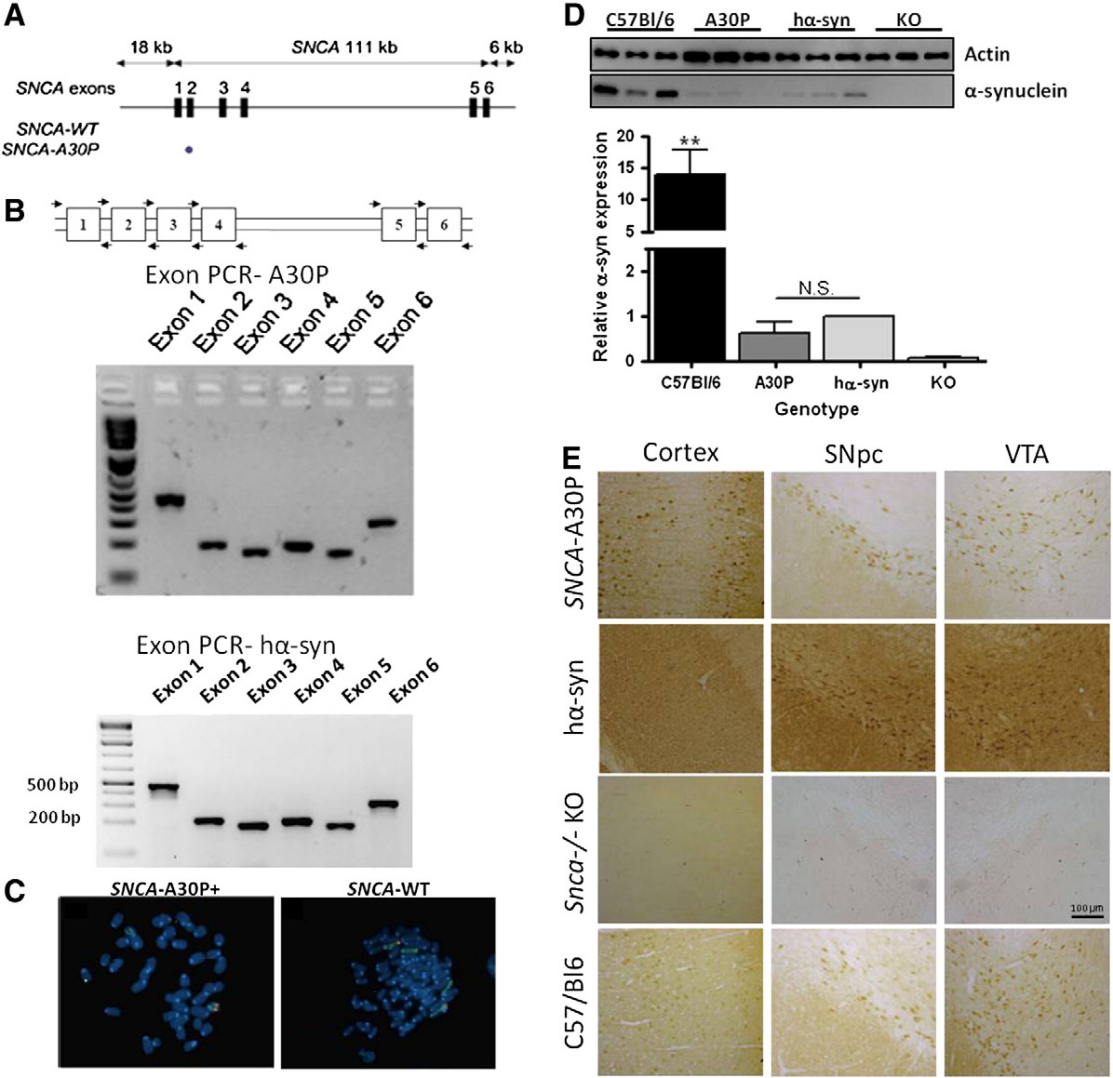
Expression of human wild-type or A30P mutant α-synuclein in *SNCA*-BAC transgenic mice. A, Schematic representation of the human *SNCA* BAC transgene constructs carrying either wild-type or A30P mutant *SNCA*. B, Confirmation of the *SNCA* BAC transgene integrity by PCR in both *SNCA* lines. Human-specific PCR primers spanning each of the *SNCA* coding exons were used (exons 1–6). C, Location of transgene integration in *SNCA*-BAC lines. Fluorescent *in situ* hybridization (FISH) confirmed a single integration site for both transgenic lines using BAC probes in combination with chromosome painting. The *SNCA-*A30P transgene integration was observed near the telomere on one copy of chromosome 15; the *SNCA*-WT (hα-syn) transgene integration occurred near the telomere on one copy of chromosome 2. D, Expression of α-synuclein was quantified by western blot of whole brain homogenates from 3 month-old *SNCA* BAC mice. α-synuclein expression was quantified relative to actin levels and normalized to expression in mice expressing wildtype human α-synuclein (hα-syn). The difference in expression between A30P and hα-syn lines is not significant. *n* = 3, **p < 0.01. E, Immunohistochemical analysis in coronal brain sections of 3-month old animals revealed α-synuclein transgene expression in the cortex, SNpc, and VTA in hα-syn and *SNCA-A30P* mice recapitulating a spatial pattern of expression almost identical to that of endogenous α-synuclein protein in wild-type C57Bl6 mice. α-synuclein expression was not seen in *Snca −/−* KO animals.

The human PD-associated *SNCA* A30P point mutation was engineered into a human genomic DNA BAC construct derived from clone PAC-27 M07 using positive/negative selection/counter-selection homologous recombination in *E. coli* as previously described (Alegre-Abarrategui et al., 2009). The 135 kb genomic DNA *SNCA* BAC insert contains the 111 kb *SNCA* locus, flanked by 18 kb of 5′ and 6 kb of 3′ genomic DNA sequence (Fig. 1A). The inclusion of 18 kb of 5′ promoter ensures the presence of the NACP-Rep1 repeat element which lies approximately 10 kb upstream of the transcriptional start site, and is an important regulatory element (Jakes et al., 1994, Ueda et al., 1993). Previous work has shown strongest promoter activity is found from constructs containing at least 6.2 kb of upstream DNA (Chiba-Falek et al., 2003, Chiba-Falek et al., 2005, Chiba-Falek and Nussbaum, 2001, Touchman et al., 2001, Xia et al., 2001). The accuracy of the recombineering was confirmed by restriction enzyme analysis and DNA sequencing. Transgenic founder animals identified as carrying intact BAC inserts following pro-nuclear injection of wild-type or A30P mutant *SNCA* BAC constructs into a C57/Bl6 background were used to establish breeding lines. The lines were backcrossed onto a mouse C57/Bl6 *Snca −/−* knockout (KO) background for a minimum of six generations to remove the endogenous α-synuclein, producing lines hemizygous for either the wild-type *SNCA* or the *SNCA-A30P* transgene on a pure C57Bl/6 *Snca −/−* background, to generate, respectively *SNCA-WT + Snca −/−* (referred to as hα-syn) or *SNCA*-*A30P* + *Snca −/−* (referred to as A30P). Importantly, back-crossing to a *Snca −/−* background avoids the previously-reported confounding effect of endogenous mouse α-synuclein expression on transgene function (Cabin et al., 2005).

The presence of all *SNCA* exons in both the transgenic hα-syn and A30P lines was confirmed by PCR using primers specific to human DNA (Fig. 1B), and the site of BAC integration for both lines was identified by fluorescence in situ hybridization (FISH) (Fig. 1C). Western blot analysis confirmed expression of the human α-synuclein protein in the brains of the hα-syn and A30P lines at similar levels; however, *SNCA* transgene expression was significantly less than endogenous mouse α-synuclein in C57/Bl6 mouse brain (Fig. 1D). Immunohistochemical analysis in coronal brain sections of hα-syn and A30P mice revealed a spatial pattern of α-synuclein expression that recapitulates the pattern of endogenous α-synuclein protein in wild-type C57/Bl6 mice. Regions showing α-synuclein protein expression include the cerebral cortex, hippocampus, striatum, and importantly the SNpc and VTA (Fig. 1E).

### Immunohistochemical analysis of α-synuclein expression in dopaminergic neurons in SNCA-BAC transgenic mice

**Fig. 2:**
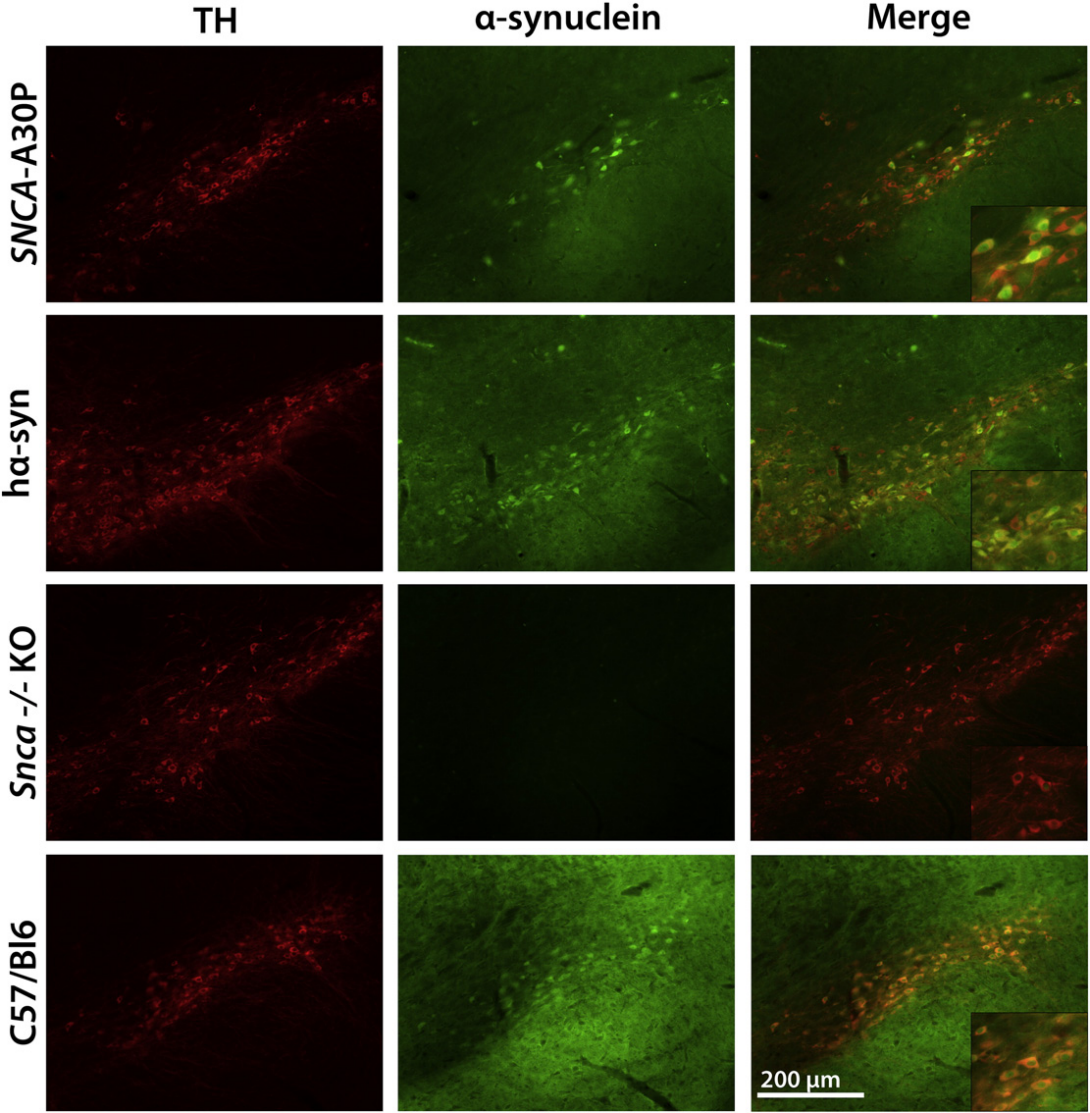
Immunohistochemical analysis of α-synuclein expression in the SNpc in *SNCA*-BAC transgenic mice. Immunofluorescence shows human α-synuclein protein expression in 3 month-old A30P and hα-syn mice that accurately recapitulates the pattern of endogenous α-synuclein expression in TH-positive neurons in the SNpc, seen as yellow cells in the merged frame and clearly visible in the high-magnification insets. Age-matched *Snca −/−* KO animals display no α-synuclein expression.

Fluorescent double-labeling for tyrosine hydroxylase (TH) and α-synuclein in neuronal slices confirmed accurate physiological expression of the α-synuclein transgene in TH-positive cells in the substantia nigra pars compacta (SNpc), the region most commonly associated with neuronal degeneration in PD (Fig. 2). Intracellularly, α-synuclein protein was observed in the axons, cell bodies and in the nucleus in A30P mice, hα-syn mice and wild-type C57/BL6 mice. This demonstrates that the presence of α-synuclein in the cell body and nucleus is a physiologically normal feature and is not a consequence of the A30P mutation. Double-label immunofluorescence staining of sections followed by cell count analysis showed that 47.6 ± 3.2% of TH + cells in the SNpc in the A30P line at 6 months of age also expressed α-synuclein (*n* = 3 mice).

### Expression of wild-type or A30P SNCA restores sensitivity to MPTP in Snca −/− mice

**Fig. 3:**
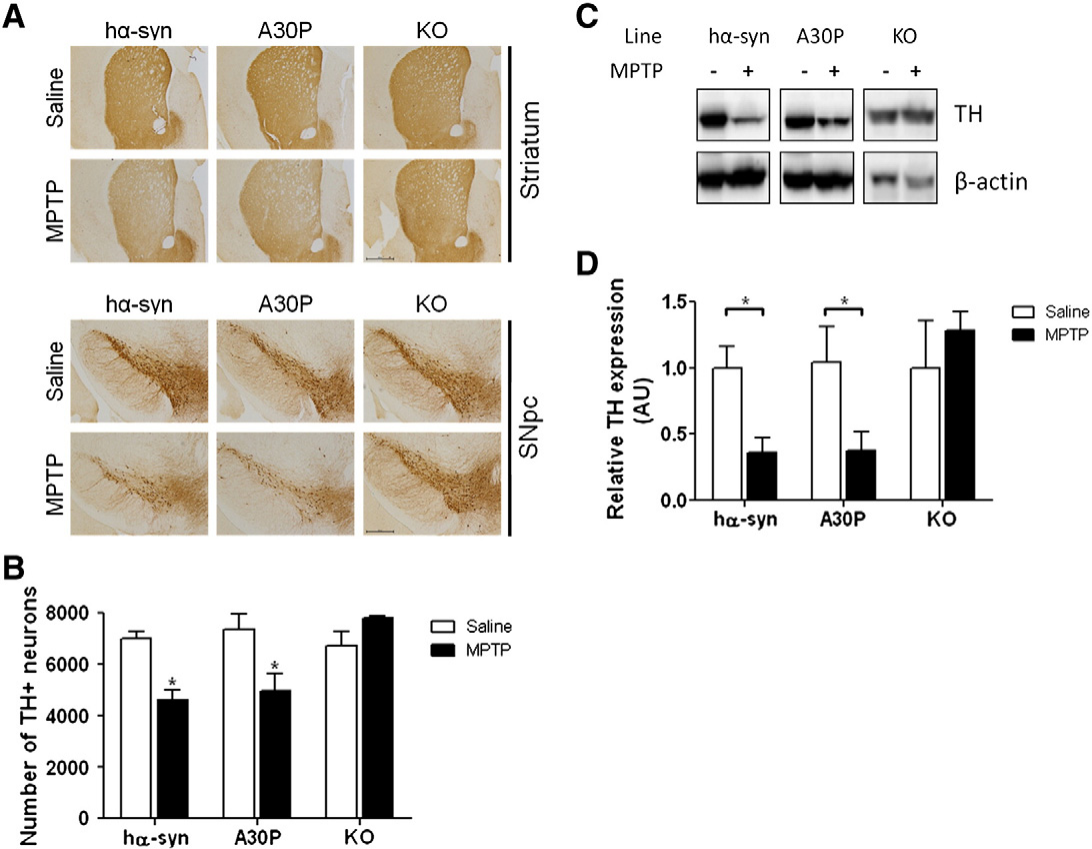
Expression of wild-type or A30P α-synuclein restores susceptibility of DA neurons in *Snca −/−* mice to MPTP in 6-month old mice. A, TH immunoreactivity in striatum and SNpc in hα-syn, A30P, and *Snca −/−* KO mice 7 days following saline or acute dosing of MPTP free base. The A30P animals are similarly susceptible to MPTP lesion as hα-syn mice, as evidenced by a reduction of TH staining in the striatum and SNpc. Representative sections are shown. B, Quantitative unbiased stereological cell counts show a 30% reduction in TH + neurons in the SNpc of hα-syn and A30P animals. The *Snca −/−* KO animals were resistant to MPTP treatment. Results represent mean ± SEM for 3 animals per genotype and treatment *p < 0.05. C, D The level of striatal TH was diminished in hα-syn and A30P animals after exposure to MPTP as measured by western blot analysis of TH levels in striatal tissue, while TH expression remained unchanged in *Snca −/−* KO animals. TH band intensity was controlled for β-actin expression and normalized, within-genotype, to saline-treated controls (*n* = 3, *p < 0.05).

We next investigated whether the expression of the wild-type or A30P mutant α-synuclein restored the sensitivity of DA neurons to the dopaminergic neurotoxin, MPTP, in otherwise resistant *Snca −/−* animals. When challenged with an acute dose of MPTP, both the hα-syn and A30P mice were found to be equally susceptible to nigral and striatal damage by histological observation of TH staining (Fig. 3A) and by unbiased stereological analysis of the SNpc (Fig. 3B) whereas the *Snca −/−* KO littermate was resistant to MPTP-induced nigrostriatal damage as previously reported (Dauer et al., 2002, Thomas et al., 2011). In the SNpc, both hα-syn and A30P animals demonstrated a 30% loss of TH + neurons in response to MPTP treatment, while there was no change in MPTP-treated *Snca −/−* KO animals as analyzed by two-way ANOVA with Bonferroni *post-hoc* tests (hα-syn: saline: 7003 ± 296, MPTP: 4626 ± 372; A30P: saline:7358 ± 599, MPTP: 4963 ± 669; KO: saline: 6735 ± 533, MPTP: 7816 ± 53; interaction between treatment and genotype: *p* = 0.0137, F_(2,10)_ = 6.79, *n* = 3). Loss of dopaminergic neuron markers in the striatum of hα-syn and A30P lines, but not in *Snca −/−* KO animals, following MPTP treatment was also confirmed by quantitative analysis of TH protein levels by western blotting in striatal lysates (Figs. 3C, D). These data show that the A30P mutant is functionally equivalent to the wild-type α-synuclein in being able to restore sensitivity to MPTP.

### A30P transgenic mice display deficits in electrically evoked release of dopamine but not norepinephrine

**Fig. 4:**
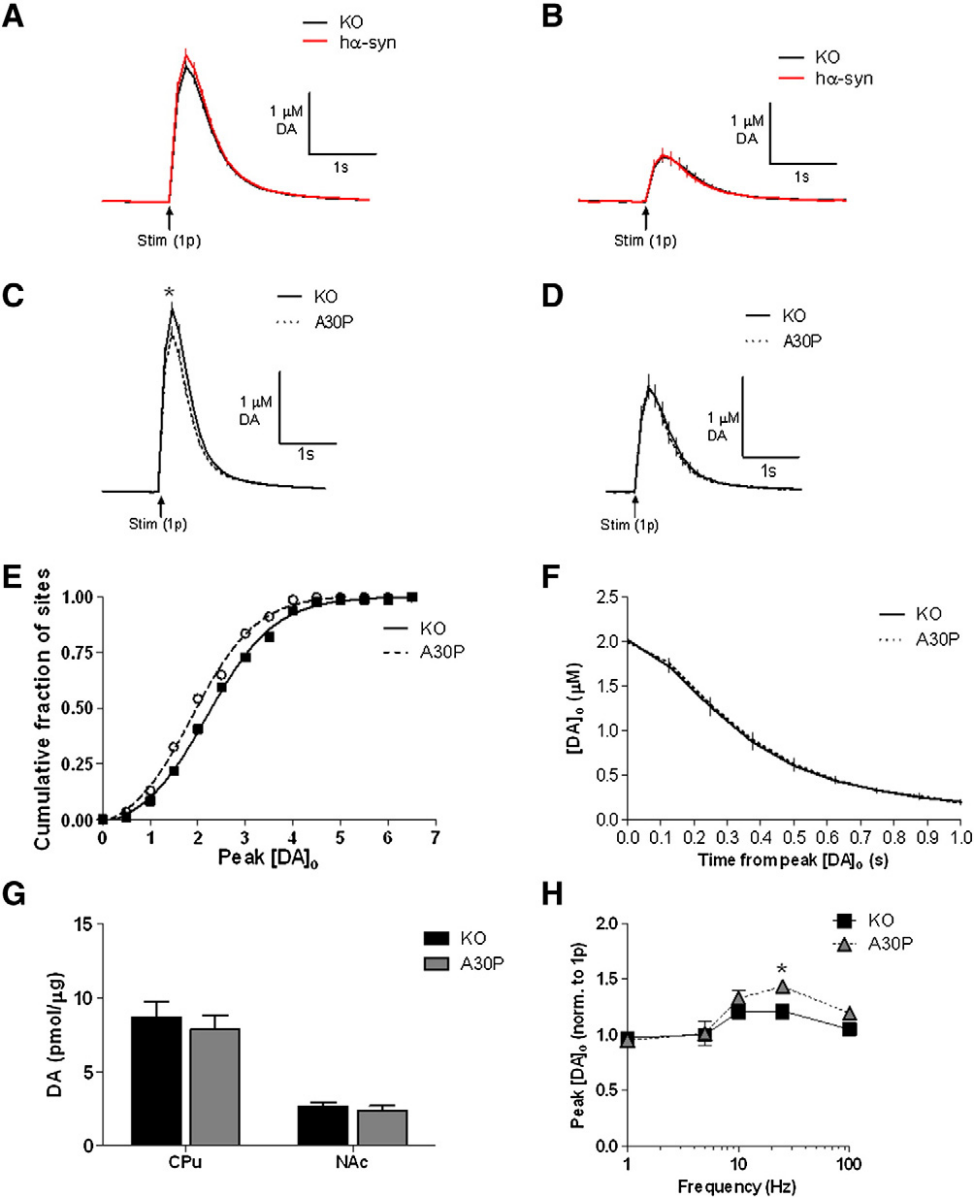
Electrically evoked dopamine transients and regulation of [DA]_o_ by firing frequency in 3-month old hα-syn, *SNCA* A30P and *Snca −/−* KO measured by FCV. A, Mean profiles of [DA]_o_*versus* time (mean ± SEM) after a single pulse (0.2 μs; arrow) in the dorsal striatum (CPu). In CPu, there is no difference in peak [DA]_o_ transiently evoked by single pulses in hα-syn mice compared to age-matched KO littermates (*p* = 0.2265, *n* = 60 (5 animals per genotype)). B, No significant differences seen in mean peak [DA]_o_ evoked by a single pulse (1p) in hα-syn transgenic mice compared to KO littermates in the NAc. (*p* = .8680, *n* = 18 (5 animals per genotype)). C, Mean profiles of [DA]_o_*versus* time (mean ± SEM) after a single pulse (0.2 μs; arrow) in the dorsal striatum (CPu). In CPu, peak [DA]_o_ transiently evoked by single pulses is slightly less in A30P than age-matched KO littermates (*p < 0.05, KO, *n* = 96; A30P, *n* = 92 (9 animals per genotype)). D, No significant differences seen in mean peak [DA]_o_ evoked by a single pulse (1p) in A30P transgenic mice compared to KO littermates in the NAc. E, Cumulative histogram of peak [DA]_o_ of evoked DA transients in the CPu in KO *vs* A30P mice for individual sampling sites. F, Comparison of the rates of decay of concentration-matched DA transients suggests that DA uptake rates are not significantly different between the two genotypes (*n* = 5, *p* > 0.05). G, No differences were observed in DA content (pmol per μg of protein) in the CPu or NAc dissected from striatal slices. Data shown represent mean ± SEM from *Snca* KO and *SNCA* A30P mouse samples (*n* = 8–10, *p* > 0.05). H, Regulation of [DA]_o_ by firing frequency in the CPu. Mean peak [DA]_o_ during 5 pulse trains (1–100 Hz) in the CPu varied with frequency in both genotypes in control conditions, with a slightly greater frequency sensitivity observed in A30P transgenic mice at 25 Hz. Data were normalized within genotype to the 1p value (*n* = 3, *p < 0.05).

We first assessed the release and re-uptake of DA in the CPu and NAc using FCV in A30P and hα-syn transgenic mice and compared to *Snca −/−* (KO) littermate controls. We have previously shown that *Snca −/−* mice show the same evoked [DA]_o_ in response to a single stimulus or a burst stimulus as wild-type mice (Senior et al., 2008) allowing us to use the *Snca −/−* KO littermates as controls. When we compared evoked [DA]_o_ in *Snca −/−* KO animals to the hα-syn animals, no differences were seen after a single stimulus pulse (1p) in the CPu or NAc (Figs. 4A, B; CPu: *Snca −/−* KO: 2.13 ± 0.098, *n* = 60 sites; hα-syn: 2.298 ± 0.099, *n* = 60 sites; 5 animals per genotype, *p* = 0.23, *t*-test: *t* = 1.216, df = 118; NAc: *Snca −/−* KO: 0.70 ± 0.1, *n* = 18 sites; hα-syn: 0.73 ± 0.11, *n* = 18 sites; 5 animals per genotype, *p* = 0.87, *t*-test: *t* = 0.1674, df = 34). However, in the CPu from A30P mice, evoked [DA]_o_ were significantly reduced (~ 13%) compared to *Snca −/−* KO after either a single stimulus pulse (1p) (*Snca −/−* KO: 2.63 ± 0.11 μM, *n* = 96 sites; A30P: 2.29 ± 0.10 μM, *n* = 92 sites; 9 animals per genotype, *p* = 0.02, *t*-test: *t* = 2.319, df = 186) (Fig. 4C), and or a burst stimulus (4 pulses, 100 Hz) (*Snca −/−* KO: 2.56 ± 0.11 μM, *n* = 94 sites; A30P: 2.27 ± 0.10 μM, *n* = 95 sites; 9 animals per genotype, *p* = 0.04, *t*-test: *t* = 2.024, df = 187) (data not shown). In the NAc, no significant differences in evoked [DA]_o_ were detected between the A30P and *Snca −/−* KO genotypes (Fig. 4D; *Snca −/−* KO: 1.51 ± 0.17 μM, *n* = 30 sites; A30P: 1.53 ± 0.17 μM, *n* = 30 sites; 5 animals per genotype, *p* = 0.92, *t*-test: *t* = 0.1021, df = 58). The decreased release in the A30P animals compared to *Snca −/−* KO in the CPu is evident in the shift to the left of the cumulative histogram of peak [DA]_o_ of evoked DA transients for individual sampling sites (Fig. 4E). In CPu, the decrease in evoked [DA]_o_ in A30P mice was not due to variation in striatal DA uptake or DA content: no significant differences were seen in the decay phases of DA transients from A30P compared to KO mice (Fig. 4F) or in DA content (Fig. 4G).

To further investigate the deficits in evoked [DA]_o_ in A30P mice the frequency sensitivity of DA release in A30P *versus* KO mice was assessed in the CPu using 5-pulse trains at frequencies ranging from 1 to 100 Hz. These stimulation frequencies span the full physiological range, and beyond, of DA neuron firing frequencies including tonic and burst-like activities. Although [DA]_o_ varied with frequency according to a slight inverted U-relationship in the CPu in both genotypes, as previously published in wild-type C57Bl/6 mice (Exley et al., 2008), the A30P animals showed a subtle but significant enhancement of frequency sensitivity (Fig. 4H). These data are consistent with a deficit in DA release probability underlying diminished release by a single pulse and a corresponding reduction in short-term depression that would enable greater release during pulse trains (Rice and Cragg, 2004).

**Fig. 5:**
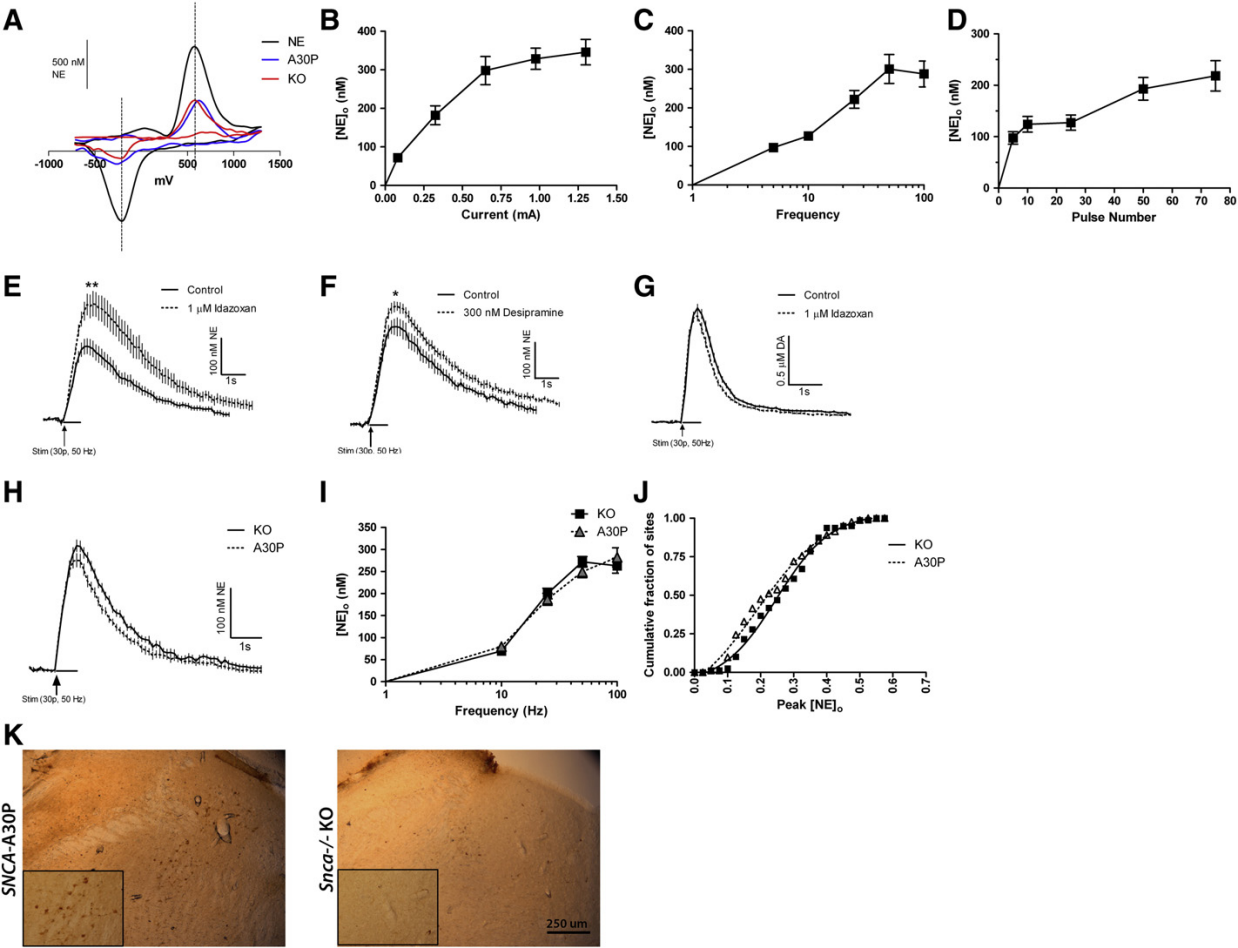
Electrically evoked noradrenergic transients and regulation of norepinephrine (NE) signals by firing frequency in 3-month old *SNCA*-A30P and *Snca −/−* KO mice measured by FCV. A, Cyclic voltammograms from a calibration using 2 μM NE (black), and 30 pulses at 50 Hz in *Snca* KO (red) and A30P (blue) slices. B, Maximal NE release as a function of current amplitude. Each data point was obtained with a 30-pulse, 50 Hz stimulation (*n* = 6 C57Bl/6 animals). C, Maximal release as a function of stimulation frequency. Each data point was obtained with a 30-pulse stimulation, and a current amplitude of 0.65 mA (*n* = 5 C57Bl/6 animals). D, Maximal release as a function of pulse number. Each data point was obtained with a stimulation frequency of 50 Hz, and current amplitude of 0.65 mA (*n* = 6 C57/Bl6 animals). E, Mean [NE]_o_*versus* time evoked by 30 pulses at 50 Hz in vBNST of C57/Bl6 mice in control conditions, in the presence of the α_2_ adrenergic antagonist, idazoxan (1 μM). Idazoxan significantly increased peak [NE]_o_ in the vBNST (*n* = 3 animals, **p < 0.01). F, Mean [NE]_o_*versus* time evoked by 30 pulses at 50 Hz in vBNST of C57Bl/6 mice in control conditions and in the presence of 300 nM desipramine. Desipramine significantly increased peak [NE]_o_ in the vBNST (*n* = 3 animals, *p < 0.05). G, Mean [DA]_o_*versus* time evoked by 30 pulses at 50 Hz in the CPu of C57/Bl6 mice in control conditions and in the presence of α_2_ adrenergic antagonist, idazoxan (1 μM). Unlike in the vBNST, idazoxan produces no change in peak [DA]_o_ in the CPu (*n* = 3 animals). H, Mean profiles of [NE]_o_*versus* time (mean ± SEM) after a 30 pulse, 50 Hz stimulation (arrow) in the ventral bed nucleus of the stria terminalis (BNST). In vBNST, a very slight 8% difference was observed in peak [NE]_o_ transiently evoked by 30 pulses at 50 Hz in A30P compared to age-matched KO littermates which was not significant (*p* > 0.05; KO, *n* = 79 sites; A30P, *n* = 82 sites (8 animals per genotype)). I, Mean peak [NE]_o_ ± SEM *vs.* frequency during 30 pulse trains (10–100 Hz) in the vBNST varied with frequency in both genotypes in control conditions. No differences in frequency sensitivity were seen in A30P transgenic mice compared to KO animals (*p* > 0.05; 8 animals per genotype). J, Cumulative histogram of peak [NE]_o_ of evoked NE transients in the vBNST in KO *vs* A30P mice for individual sampling sites. K, Immunohistochemical analysis in coronal mouse sections confirmed α-synuclein transgene expression in the LC in *SNCA-A30P* mice; α-synuclein expression was not seen in *Snca −/−* KO animals.

To investigate if the release deficit observed in the A30P mice is seen at sites of release of other monoamines we next explored the regulation of NE release in the vBNST. The vBNST is a site of relatively dense innervation by NE but not DA axons, unlike the dorsal BNST where DA predominates (Kilts and Anderson, 1986). Given the potential overlap in catecholamine distribution and the similarity in DA and NE voltammograms detected with FCV, it was important to characterize the electrochemical signals detected with pharmacology. Evoked cyclic voltammograms in each genotype had current peak potentials characteristic of those of 2 μM NE (or DA) in calibration (Fig. 5A). We first optimized the stimulation protocol for subsequent studies using C57Bl/6 wild-type animals and an inter-stimulation interval of 7 min (Miles et al., 2002). During a stimulus train of 30 pulses at 50 Hz, the concentration of catecholamine varied with current up to an asymptotic value reached at approximately 0.65 mA (Fig. 5B) The concentration of evoked catecholamine also increased with frequency during 30-pulse trains up to a maximum at 50 Hz (Fig. 5C) and at a stimulation frequency of 50 Hz, varied with pulse number (Fig. 5D). A stimulus protocol of 30 pulses at 50 Hz (and 0.65 mA) was selected for subsequent studies to deliver good signal-to-noise, and with minimal loss of release over time (data not shown).

To identify the catecholamine detected as NE rather than DA, we explored whether the catecholamine signal in vBNST was modified by an NE transporter uptake inhibitor, desipramine, and by an α_2_ adrenergic receptor antagonist, idazoxan. In the vBNST, either desipramine (300 nM) or idazoxan (1 μM) significantly increased [NE]_o_ evoked by a 30-pulse train at 50 Hz (idazoxan: *n* = 15–20 sites, 3 animals; *p* = 0.0047, *t*-test: *t* = 3.051, df = 30, Fig. 5E; desipramine: *n* = 8–10 sites, 3 animals; *p* = 0.0247, *t*-test: *t* = 2.496, df = 15, Fig. 5F). Conversely, in CPu where evoked signals are attributab. to DA (*e.g.* Cragg et al., 1997), idazoxan (1 μM) had no effect on [DA]_o_ evoked by an identical stimulus train (*n* = 11–13 sites, 3 animals; Fig. 5G). It has also previously been shown that 300 nM desipramine does not affect [DA]_o_ evoked with these protocols in the CPu (Cragg et al., 1997). Together these data suggest that the evoked catecholamine signals in vBNST are attributab. to NE rather than DA.

We then compared NE transmission in vBNST of A30P *versus Snca −/−* KO mice. In the vBNST of A30P slices, [NE]_o_ evoked by a 30-pulse train at 50 Hz were not significantly less than those detected in *Snca −/−* KO mice (KO: 0.27 ± 0.01 μM, *n* = 79 sites; A30P: 0.25 ± 0.01 μM, *n* = 82 sites; 8 animals per genotype, *p* = 0.20, *t*-test: *t* = 1.278, df = 159) (Fig. 5H). The frequency sensitivity of NE release in A30P and KO mice during 30-pulse trains varied with frequency but not genotype (*n* = 42, main effect of genotype F_(1,405)_ = 0.03, *p* = 0.87, 2-way ANOVA; Fig. 5I). No difference was observed between genotypes in the NE content in the vBNST dissected from slices (A30P: 18.5 ± 4.8 pmol/ng, KO: 17.8 ± 6.9 pmol/ng, *n* = 5 animals per genotype, *p* = 0.94, *t*-test: *t* = 0.076, df = 7). Finally, we confirmed expression of the α-synuclein transgene in the LC of the A30P animals, which was absent in the *Snca −/−* KO mice (Fig. 5K).

### Aged A30P mice do not display catecholaminergic neuron loss nor α-synuclein neuropathology

**Fig. 6:**
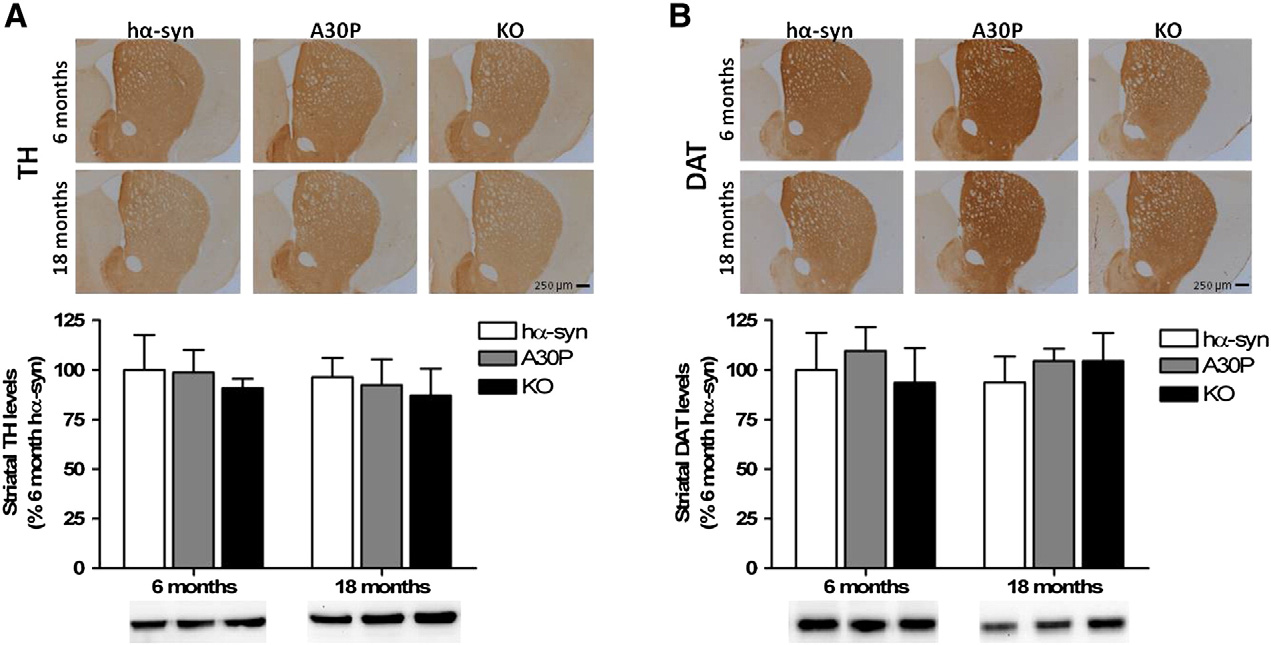
Immunohistochemical and western blot analysis of striata in aged (18-month old) hα-syn, A30P, and *Snca −/−* KO mice. A, TH immunoreactivity was unchanged at 18 months of age. Western blot analysis of striatal lysates from hα-syn, A30P, and *Snca −/−* KO mice show also no differences in TH expression with age. B, DAT immunoreactivity was unchanged at 18 months of age. Similarly, western blot analysis of striatal lysates from hα-syn, A30P, and *Snca −/−* KO mice does not demonstrate a reduction in DAT expression with age. Analysis was performed on 3 animals per genotype at each age. Representative sections are shown. Columns represent percentage change from the 6-month old hα-syn control. Results represent the mean ± SEM for three animals per genotype at each age. For western blot analysis, 10 μg protein was loaded per lane.

**Fig. 7:**
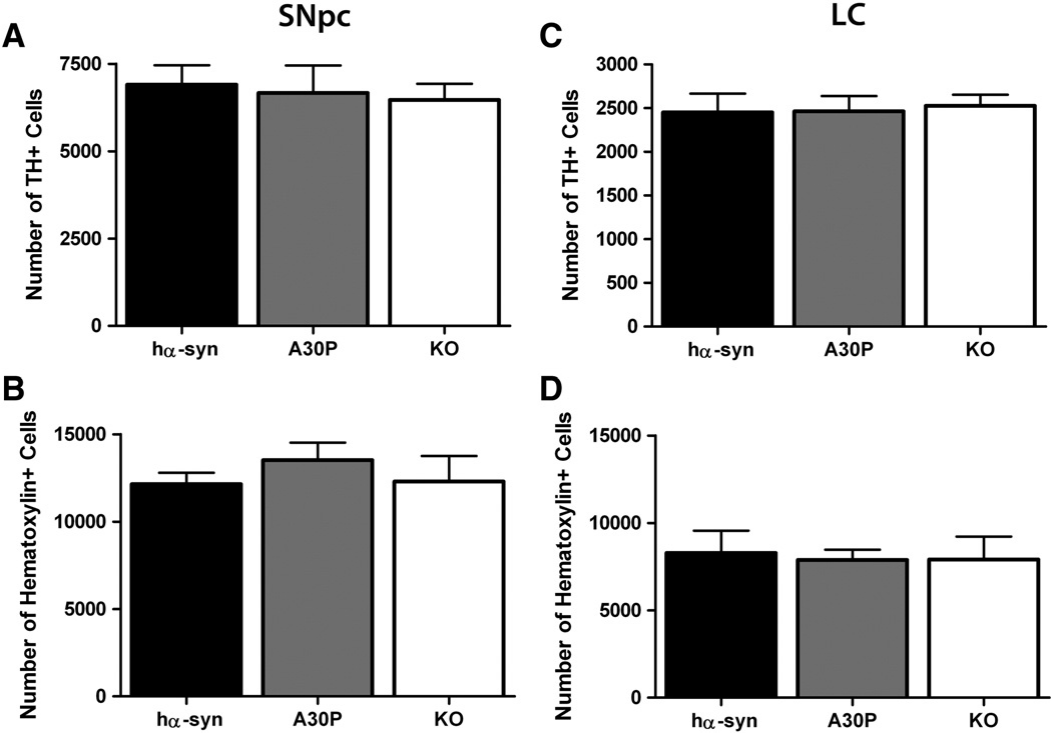
Stereological counts of SNpc and LC in aged hα-syn, A30P, and *Snca −/−* KO mice. A, B No differences were seen in TH-positive cells (A) or hematoxylin-positive (B) in the SNpc at 18 months of age in A30P animals compared with hα-syn and KO animals. C, D, Similarly, no differences were observed TH cell number (C) or hematoxylin-positive cell numbers (D) in the LC at 18 months of age between the three genotypes. Results represent the mean ± SEM for 5 animals per genotype at each age, *p* > 0.05.

No changes in striatal TH or dopamine transporter (DAT) expression were observed in the A30P mice at 6 or 18 months of age compared to age-matched hα-syn and *Snca −/−* KO animals, as measured by immunohistochemical analysis and western blot (Fig. 6). To investigate for loss of catecholaminergic neurons with age, unbiased stereological cell counts were performed on TH/hematoxylin stained cells from the SNpc and LC of A30P, hα-syn, and *Snca −/−* KO mice. At 18 months of age, no change in the number of nigral TH-positive (hα-syn: 6340 ± 709; A30P: 6668 ± 786; KO: 6467 ± 464; *p* = 0.89) (Fig. 7A) or hematoxylin-stained (hα-syn: 12,147 ± 647; A30P: 14,248 ± 1067; KO: 12,290 ± 1471; *p* = 0.67) cells (Fig. 7B) was observed. Similarly, there were no changes in the number of TH-positive (hα-syn: 2257 ± 249; A30P: 2463 ± 175; KO: 2526 ± 128; *p* = 0.95) (Fig. 7C) or hematoxylin stained (hα-syn: 8276 ± 1284; A30P: 7884 ± 583; KO: 7908 ± 1317; *p* = 0.96) (Fig. 7D) cells in the LC. Finally, a count of TH-positive neurons in sub-regions of the SNpc (dorsal *vs* lateral *vs* medial and also caudal *vs* rostral), based on Fu et al. (2012) to investigate any region-specific loss which may be masked when considering the SNpc as a whole, confirmed no differences in TH-positive neurons between genotypes at 18 months (data not shown).

**Fig. 8:**
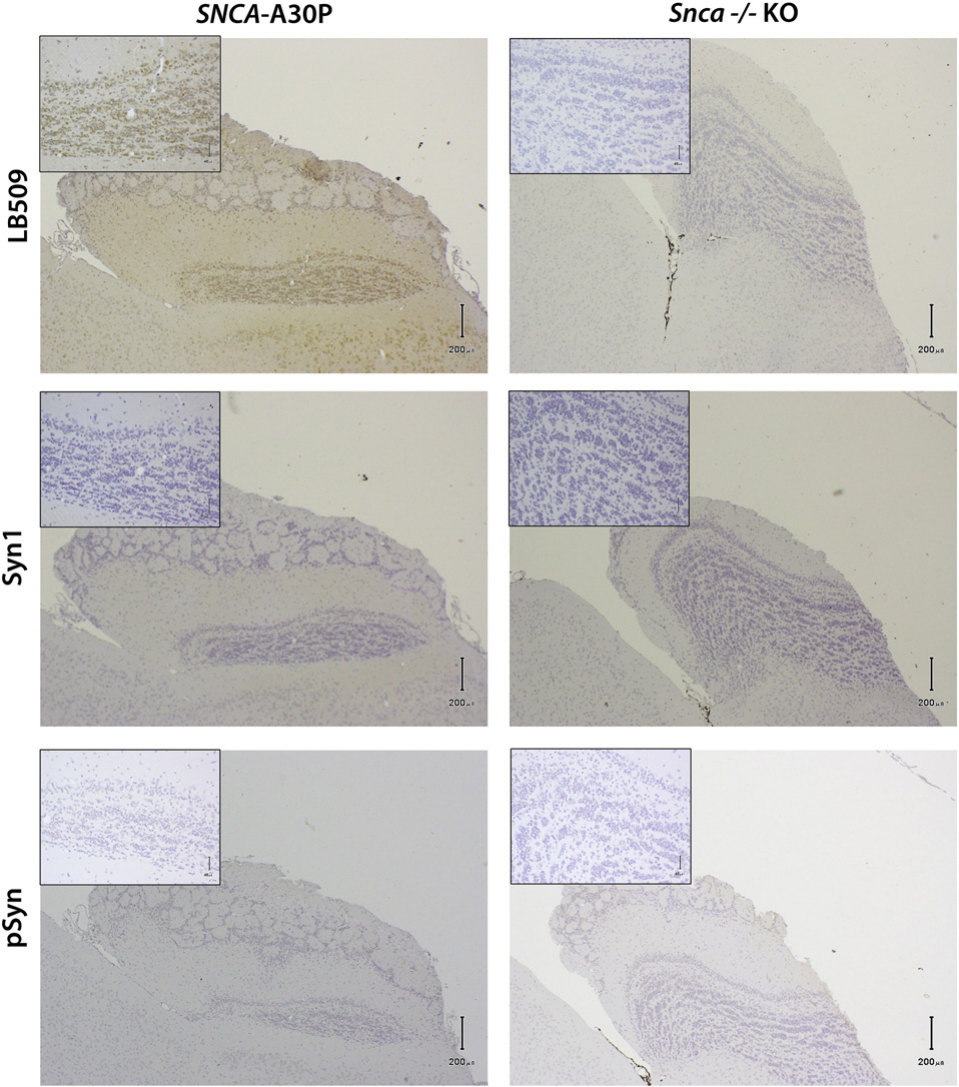
Absence of synuclein pathology in 23-month old A30P transgenic animals. A diffuse cytosolic α-synuclein immunostaining was seen in neuronal cells of the anterior olfactory nucleus and granular cell layer of the olfactory bulb with antibody LB509 (A), but not by Syn-1 (C), or pSyn #64 against the phosphorylated α-syn (E). Magnification in A-F × 40, inserts show higher-power magnified image × 200.

We performed immunohistochemistry of A30P mice and their *Snca −/−* littermate controls in paraffin-embedded sections to look for abnormal accumulation or aggregation of α-synuclein. In A30P mice at 18–23 months of age the antibody LB509 revealed a diffuse cytosolic α-synuclein immunostaining in neuronal cells of the anterior olfactory nucleus and granular cell layer of the olfactory bulb (Figs. 8A, B) and in some pyramidal neurons in the CA2-3 region of the hippocampus (data not shown). However, no immunostaining was detected by Syn-1 or by pSyn#64, which detects phosphorylated α-synuclein (Figs. 8C–F). No α-synuclein accumulation was seen in the TH-positive neurons of the midbrain or in the spinal cord (data not shown). Immunohistochemical staining for a common constituent in several types of disease-associated inclusions, p62 ubiquitin binding protein/sequestosome 1, revealed no pathological protein-aggregations (data not shown). GFAP immunohistochemical staining did not show any astrogliosis and Iba-1 staining showed no increased microglial activation in the A30P mice compared to *Snca −/−* KO and hα-syn mice (data not shown).

### A30P mice exhibit a mild behavioral phenotype

**Fig. 9:**
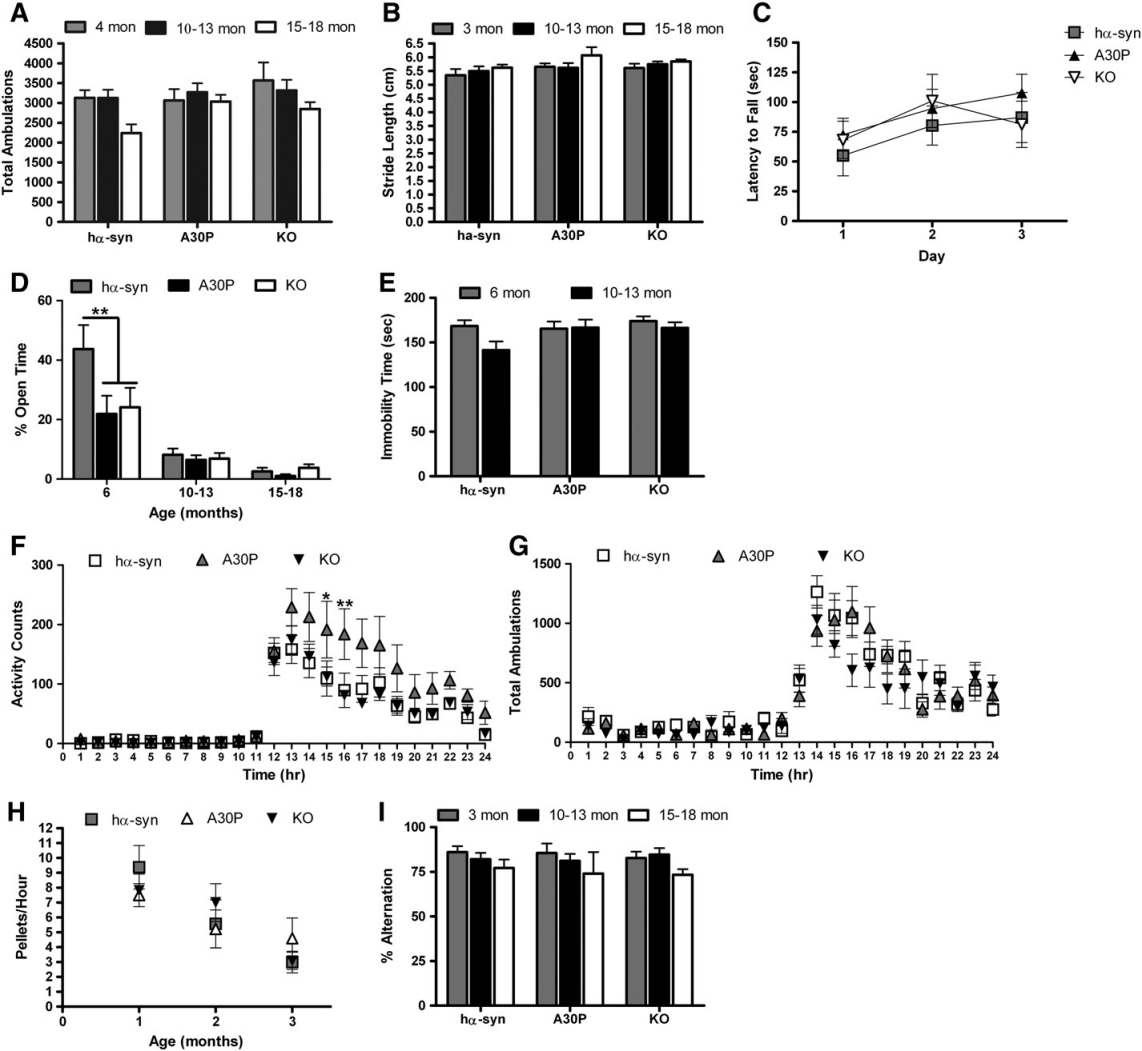
A30P mice display a very mild behavioral phenotype. A, A30P mice have similar total locomotor activity at all ages when introduced into a novel environment as compared to age-matched hα-syn and *Snca −/−* KO controls. Results represent the mean number of ambulations ± SEM for 10–13 mice per genotype. B, No deficits in forepaw stride length were apparent at 3 months or 10–13 months of age in A30P mice, compared to age-matched hα-syn and *Snca −/−* KO controls. Results represent average stride length (cm) ± SEM for 8–10 animals per genotype. C, No differences were seen in motor learning or performance on the rotarod at 18 months of age. Results represent latency to fall (sec) ± SEM for 4–10 animals per genotype. D, At 6 months of age, the hα-syn mice spent a greater percentage of their time in the open arms of the elevated plus maze as compared to age-matched A30P and *Snca* KO animals over the 5 min test period. All genotypes display an increased anxiety-like phenotype with age. Results represent the mean percentage of time spent in the open arms ± SEM for 10–13 mice per genotype. **p < 0.01 E, All genotypes had similar immobility times in the tail suspension test, which remains unchanged with age. Results represent mean time (s) ± SEM for 10–13 mice per genotype. F, A30P mice display increased wheel running during hours 16–17 of their light–dark cycle compared to hα-syn and *Snca −/−* KO mice at 12 months of age. Results represent total activity counts ± SEM for 5 animals per genotype *p < 0.05, **p < 0.01. G, A30P mice display normal circadian activity levels compared to hα-syn and KO animals at 12 months of age. Results represent total ambulations ± SEM for 8 animals per genotype. H, A30P mice do not display differences in stool frequency compared to age-matched hα-syn and *Snca −/−* KO controls. Results represent average stool frequency ± SEM for 5–11 animals per genotype. I, A30P mice do not display deficits in spontaneous alternation as measured by the T-maze at 3, 10–13, or 15–18 months of age when compared to age-matched hα-syn or *Snca* KO mice. Results represent the mean alternation ± SEM for 10–13 animals per genotype at each age.

We tested the A30P mice at several timepoints between 3 and 18 months of age for a range of motor and non-motor phenotypes associated with PD. Before testing the animals for PD phenotypes, the mice were first assessed for general sensory deficits which might interfere with behavioral testing. A30P mice showed no deficits in response to tactile stimulation, mild irritation/burning sensations stimulated by ammonia, or quinine-induced taste aversion as compared to hα-syn and *Snca −/−* KO controls (data not shown). Aging A30P mice up to 18 months of age were tested for the presence of motor phenotypes. Animals were tested using novelty-induced locomotor activity, forepaw stride length, and rotarod. The A30P mice did not demonstrate any reductions in novelty-induced activity when compared to age-matched hα-syn and *Snca −/−* KO mice (*n* = 5–10, interaction effects of age and genotype: F_(4,76)_ = 0.72, *p* = 0.58) (Fig. 9A). When assessed by forepaw stride length, no differences were detected between genotypes at any age (*n* = 5–10, interaction effect of age and genotype: F_(4,78)_ = 0.34, *p* = 0.85) (Fig. 9B). Likewise, when tested on an accelerating rotarod paradigm, 18 month A30P animals displayed no differences in motor learning or performance when compared to age-matched hα-syn and *Snca −/−* KO animals, as analyzed by repeated measures two-way ANOVA with Bonferroni *post hoc* test (*n* = 4–10, main effect of genotype: F_(2,17)_ = 0.29, *p* = 0.75) (Fig. 9C).

Finally, the A30P animals were subjected to behavioral assays to test for non-motor symptoms associated with PD. Non-motor PD symptoms we wished to model include neuropsychiatric disorders such as anxiety and depression, gastrointestinal dysfunction, cognitive impairments, and sleep disturbances. The A30P and KO mice were shown to exhibit an anxiety-like phenotype by quantifying time spent in the open-arm of the elevated plus maze compared to the hα-syn animals at 6 months of age (main effect of genotype: F_(2, 75)_ = 31.36, *p* < 0.001; interaction effects of age and genotype: F_(4,75)_ = 2.25, *p* = 0.0715; 2-way ANOVA with Bonferroni *post hoc* analysis). However, all genotypes showed an increase in anxiety with age (*n* = 10–13, main effect of age: F_(2,75)_ = 2.66, *p* = 0.0763, 2-way ANOVA with Bonferroni *post hoc* analysis) (Fig. 9D). Animals were tested for altered behavior in the tail suspension test. At both 6 months and 10–13 months of age, there was no difference found between the immobility times of hα-syn, A30P, and *Snca −/−* KO in the tail suspension test (*n* = 10–13, interaction effects of age and genotype: F_(2,51)_ = 1.62, *p* = 0.208, 2-way ANOVA with Bonferroni *post hoc* analysis) (Fig. 9E).

When tested for differences in circadian rhythms, no significant differences were observed in the A30P animals compared to *Snca −/−* KO or hα-syn in activity during the light phase, acute response to light, re-entrainment to new light–dark cycles, or activity during constant light using methods and analyses as previously described (Oliver et al., 2012) (data not shown). However, the A30P animals were more active in wheel running during the normal 12 h–12 h normal light–dark cycle, showing a trend for increased activity throughout the dark phase, reaching statistical significance, as analyzed by repeated measures two-way ANOVA with Bonferroni *post hoc* test, during hours 4 and 5 of darkness (hours 15 and 16, *n* = 5, main effect of genotype: F_(2,12)_ = 3.52, *p* = 0.0627, interaction effect of time and genotype: F_(46,276)_ = 1.73, *p* = 0.004; Fig. 9F). Interestingly, this effect related specifically to wheel running and not activity in the dark *per se* as it was not seen during dark phase activity when mice were housed in locomotor cages and activity measured over 24 h periods of normal 12-h light/dark cycle (12:12 LD), as analyzed by repeated measures two-way ANOVA with Bonferroni *post hoc* test (Fig. 9G; *n* = 8, main effect of genotype: F_(2,21)_ = 0.64, *p* = 0.5353).

To study prevalence of a specific GI disturbance, constipation, in the A30P mice, all three genotypes of mice were behaviorally examined for stool frequency at 4, 10–13, and 15–18 months of age. Additionally, stool was analyzed for water content and dry weight at these timepoints. Stool frequency was unaltered in the A30P animals; all three genotypes have decreased stool frequency with age (*n* = 5–8, interaction effects of age and genotype: F_(4, 65)_ = 0.88, *p* = 0.479, 2-way ANOVA with Bonferroni *post hoc* analysis) (Fig. 9H). Similarly, no differences were found between genotypes in the dry weight of stool or stool water content at any age (*n* = 5–8, interaction effects of age and genotype for dry weight: F_(4,64)_ = 0.54, *p* = 0.70; interaction effects of age and genotype for water content: F_(4,62)_ = 0.44, *p* = 0.78, 2-way ANOVA with Bonferroni *post hoc* analysis, data not shown). The presence of cognitive deficits in the A30P animals was tested for by measuring spontaneous alternation in the T-maze. At 3 months, 10–13 months, and 15–18 months of age, the A30P mice display no deficits in spontaneous alternation when compared to age matched hα-syn and *Snca −/−* KO mice (*n* = 10–13, interaction effects of age and genotype: F_(4,77)_ = 0.25, *p* = 0.91, 2-way ANOVA with Bonferroni *post hoc* analysis) (Fig. 9I).

## Discussion

Here, we have exploited bacterial artificial chromosome (BAC) technology to create transgenic mouse lines to investigate the region-specific role of α-synuclein and the effect of the A30P mutation on dopamine neurotransmission. We show that expression of the A30P mutant form of α-synuclein with a correct spatial pattern results in dysregulation of dopamine neurotransmission in the dorsal, but not ventral, striatum. We propose this model recapitulates the very earliest pre-symptomatic region-specific dysfunction of the nigrostriatal dopamine system in PD.

Transgene expression in BAC mice is controlled by endogenous gene regulatory elements in genomic DNA, resulting in physiological protein expression. The inclusion of 18 kb of 5′ promoter ensures the presence of the NACP-Rep1 repeat element which lies approximately 10 kb upstream of the transcriptional start site, and is an important regulatory element (Jakes et al., 1994, Ueda et al., 1993). The hα-syn line and A30P transgenic lines express the protein at a similar level, allowing a direct comparison between their function on a *Snca −/−* background. The use of the *Snca −/−* background avoids the previously-reported confounding effect of endogenous mouse α-synuclein expression on transgene function (Cabin et al., 2005). Both transgenes have a regional pattern of α-synuclein expression which is very close to that of endogenous mouse α-synuclein in wild-type mice, with expression in regions that include the SNpc, albeit at a lower level than endogenous α-synuclein. In a recent study, transgenic mice expressing human wildtype α-synuclein, or the A30P mutated form, were created using a PAC containing the entire human *SNCA* gene, which resulted in modest levels of transgenic protein (1.3 to 2-fold compared to endogenous mouse *Snca*) (Kuo et al., 2010). Taken together, these results confirm that the BAC transgenic approach avoids the considerable overexpression of α-synuclein transgene seen when using cDNA-based transgenic models.

Previous studies have developed transgenic mouse models over-expressing human A30P α-synuclein that exhibit a range of pathologies and behaviors (Gomez-Isla et al., 2003, Lee et al., 2002, Matsuoka et al., 2001, Neumann et al., 2002, Yavich et al., 2005). Such studies have demonstrated that A30P α-synuclein expressed under the PrP or Thy-1 promoters exhibit varied motor abnormalities (Gomez-Isla et al., 2003, Neumann et al., 2002, Rathke-Hartlieb et al., 2001, Yavich et al., 2005). When tested for non-motor deficits, results have differed based on promoter; animals expressing A30P α-synuclein from the Thy-1 promoter display cognitive deficits on the Morris water maze, while animals made using the PrP promoter do not (Freichel et al., 2007, Gomez-Isla et al., 2003). A homozygous double-transgenic PAC-A30P model shows decreased stool frequency and colonic motility time, but not hyposmia or autonomic dysfunction (Kuo et al., 2010).

The range of pathologies and behaviors observed in previous A30P models may be due to overt synuclein toxicity resulting from high levels of expression rather than the effects of the mutation on the dopaminergic system or on behavioral phenotypes relevant to PD. In the present study the alterations in dopamine neurotransmission in *SNCA*-A30P mice were accompanied by a mild increase in wheel-running activity during the dark phase, a phenotype previously associated with deficits in the dopaminergic system (Knab et al., 2009, Knab et al., 2012). No significant differences were seen in anxiety, circadian rhythm, depressive-like behavior, gastrointestinal function, cognition, or motor phenotypes when tested against age-matched *Snca −/−* KO and hα-syn controls.

### α-Synuclein specifically regulates dopamine release in dorsal striatum

Our laboratory has previously shown that while *Snca −/−* KO animals do not display differences in electrically evoked [DA]_o_ in the CPu as compared to wildtype C57Bl/6 mice, mice that are either α/γ-synuclein double-null or α/β/γ-synuclein triple-null do have enhanced levels of electrically evoked DA release (Anwar et al., 2011, Senior et al., 2008). Conversely, A30P mice here displayed a 13% reduction in evoked [DA]_o_ in the CPu when compared to age-matched *Snca* KO mice. Since no differences between the *Snca*−/− KO and hα-syn mice were apparent, and the A30P and wild-type α-synuclein are expressed at comparable levels, the changes in DA transmission in A30P mice are specific to the presence of the A30P point mutation and not the human transgene. Mice lacking α-synuclein have been shown to exhibit a mild reduction in striatal tissue levels of DA (Abeliovich et al., 2000, Cabin et al., 2002) although this was not confirmed by a third study (Specht and Schoepfer, 2001). The change in evoked [DA]_o_ was not seen in the NAc, and hence appears to be specific to nigrostriatal DA tracts which are preferentially vulnerable in PD. The enhanced frequency sensitivity of DA release seen in the CPu of the A30P mice together with the reduction in release by a single pulse but the lack of change in DA content, suggests that DA release probability is reduced in this genotype. A similar modification to frequency sensitivity of [DA]_o_ has recently been identified in CPu for mice that overexpress a different human *SNCA* mutation, A53T (in a PrP promoter model) (Platt et al., 2012). These effects may represent an important change seen in synuclein-mediated PD pathophysiology. These changes in DA transmission contrast, however, with those reported for exocytosis of glutamate (Nemani et al., 2010) for which the A30P mutation decreases the ability of α-synuclein to inhibit exocytosis from cultured hippocampal neurons. This contradiction emphasizes the critical importance of undertaking studies in the neurotransmitter system of most relevance to a disease.

Given that PD pathology is not restricted to the dopaminergic system, we also examined evoked [NE]_o_ in the ventral bed nucleus stria terminalis (vBNST). Synuclein accumulation and Lewy body formation occur early in structures highly prone to neurodegeneration, including the LC; LC degeneration occurs and can be more severe than nigral degeneration (Braak et al., 2002, Braak et al., 2003, Fernagut and Chesselet, 2004, Rye and DeLong, 2003). Previous FCV work has demonstrated that NE can act as a volume transmitter in the vBNST, but NE release has not been clearly distinguished from DA (Kilts and Anderson, 1986, Miles et al., 2002). The effects of the α_2_ adrenergic antagonist, idazoxan, and the NET inhibitor, desipramine, on voltammetric signals in vBNST strongly suggest that voltammetric catecholamine signals were attributab. to NE rather than DA. We found that evoked [NE]_o_ or their frequency sensitivity were not different in A30P animals compared to age-matched *Snca −/−* KO controls confirming the region-specific role of α-synuclein on neurotransmission.

### The A30P α-synuclein mutation in PD

The α-synuclein A30P point mutation causes very rare forms of autosomal dominant PD (Kruger et al., 1998, Kruger et al., 2001). Compared to the A53T-affected patients, A30P patients are thought to exhibit a milder phenotype and later age of onset (Kruger et al., 2001), although the first *post-mortem* examination of an A30P PD patient brain recently showed strong neuropathological similarities to sporadic PD, with the A30P pathology being more severe (Seidel et al., 2010). The A30P α-synuclein mutation has been shown to prevent α-synuclein binding vesicular membranes and to promote oligomerization rather than fibrillization (Conway et al., 2000, Jensen et al., 1998). When we challenged the hα-syn and A30P mice with an acute dose of MPTP both the wild-type and A30P α-synuclein transgenes restored susceptibility to the otherwise resistant *Snca −/−* line, supporting the concept that the lipid binding ability of α-synuclein does not mediate MPTP toxicity and that A30P α-synuclein is active and able to fully complement the *Snca −/−* KO background (Dauer et al., 2002, Nemani et al., 2010). Previously, wildtype human α-synuclein and A53T mutated human α-synuclein have been shown to restore MPTP susceptibility in *Snca −/−* KO mice (Thomas et al., 2011). Here, we have demonstrated that human A30P synuclein is also able to restore neuronal vulnerability to MPTP *in vivo*.

The A30P mutation has been shown to relieve the inhibitory effect of α-synuclein on synaptic vesicle exocytosis in glutamatergic hippocampal neurons *in vitro* (Nemani et al., 2010), and it has been suggested that the inability of A30P-mutated α-synuclein to bind lipid membranes may abolish synaptic localization rendering it inactive (Fortin et al., 2004, Nemani et al., 2010). This phenomenon in non-dopaminergic neurons may be due to a number of factors including elimination of α-synuclein synaptic localization, failure to undergo structural transition, or loss of membrane interaction (Fortin et al., 2004, Jo et al., 2002, Outeiro and Lindquist, 2003). We observe that the A30P mutation affects DA neurotransmission in the nigrostriatal DA neurons projecting from the SNpc to the dorsal striatum (CPu), but not in mesolimbic DA neurons projecting from the VTA to the ventral striatum and NAc, suggesting that A30P α-synuclein may act through a gain of function in the dopaminergic nigrostriatal tract in a highly region-specific manner. Its outcome is opposite to that of deletion of α/β/γ-synuclein, and with matching region specificity (Anwar et al., 2011).

## Conclusions

Our *SNCA-*A30P BAC mouse model reflects region-specific changes seen in the dopaminergic system as a result of dysfunctional α-synuclein. Previous α-synuclein transgenic mouse models employed strong heterologous promoters, resulting in high levels of α-synuclein targeted to specific areas of the brain. Overexpression of α-synuclein (ranging up to 30-fold) constitutes a useful model of α-synuclein toxicity, but has limited physiological relevance for studying the effects of α-synuclein in PD. Additionally, regions with the most severe pathology in previous α-synuclein transgenic mice, such as motor neurons and spinal cord (Giasson et al., 2002, van der Putten et al., 2000), do not correlate with the typical regions of preferential pathology observed in PD. Taken together, these complications in previous A30P models have potentially occluded the mechanisms that represent those occurring in PD where expression levels and patterns are more moderate. BAC-based transgenic models allow for cell-specific and developmentally-appropriate expression closely recapitulating the native spatial distribution of expression. The subtle changes in dopaminergic neurotransmission observed in the A30P BAC model, despite the absence of α-synuclein-immunoreactive Lewy body-like inclusions, suggest aberrations in dopamine physiology make an important contribution to PD in the absence of protein aggregation pathology.
